# Liquid Processed Nano As_4_S_4_/SWCNTs Composite Electrodes for High-Performance Li-Ion and Na-Ion Battery Anodes

**DOI:** 10.1021/acs.energyfuels.4c03525

**Published:** 2024-10-15

**Authors:** Mark McCrystall, Cian Gabbett, Harneet Kaur, Tian Carey, Jose Munera, Lee Gannon, Cormac Mc Guinness, Valeria Nicolosi, Jonathan N. Coleman, Bharathi Konkena

**Affiliations:** † School of Physics, CRANN & AMBER Research Centres, Trinity College Dublin, Dublin D2 D02 K8N4, Ireland; ‡ School of Chemistry, CRANN & AMBER Research Centres, Trinity College Dublin, Dublin D2 D02 W9K7, Ireland

## Abstract

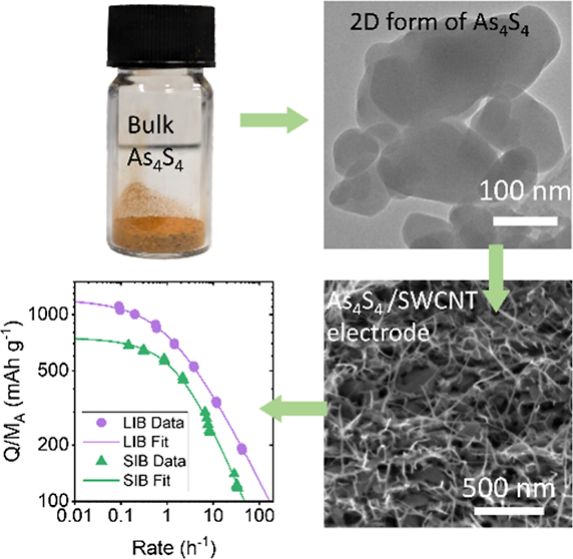

The liquid-phase exfoliation process has been successfully applied to nonlayered materials to produce quasi-2D nanoplatelets. A slight variation in bonding anisotropy in the starting material can result in the formation of 2D platelet-shaped particles with a relatively low aspect ratio. This advancement offers a promising strategy to create 2D materials from previously unexplored materials. In this study, we investigate the liquid-phase exfoliation of arsenic sulfide (As_4_S_4_), an intriguing nonlayered van der Waals material. The liquid exfoliation process generates highly disordered, low aspect ratio quasi-2D platelets. These As_4_S_4_ flakes can be easily mixed with carbon nanotubes to create nanocomposite anodes, which are appropriate for use in both Li-ion and Na-ion batteries eliminating the need for extra binders or conductive additives. The As_4_S_4_/SWCNT electrodes exhibit impressive low-rate capacities of 1202 mA h g^–1^ at 0.1 A g^–1^ for Li-ion cells and 753 mA h g^–1^ at 0.05 A g^–1^ for Na-ion cells, along with commendable cycling stability over more than 300 cycles. Detailed quantitative rate assessment clearly shows that these electrodes are limited by solid-state diffusion and emphasizing the possibility of reaching a capacity that comes close to the theoretical value which confirms the near full utilization of the active material.

## Introduction

The most commonly used energy storage technology for portable electronics is lithium-ion batteries (LIBs), which are now central to the electric vehicle industry.^1−3^ However, the growing demand for energy storage, especially in large-scale uses like grid-scale storage and electric vehicles, has sparked greater attention to alternative battery chemistries like sodium-ion batteries (SIBs).^4,5^ SIBs are of particular interest due to the abundance and cost-effectiveness of sodium, as well as the similarities in the fundamental chemistry between lithium-ion (Li-ion) and sodium-ion (Na-ion) systems.^6,7^ In the case of LIBs, new anode materials, such as silicon-based anodes,^8^ have been investigated to improve energy density due to their significant theoretical specific capacity of 3579 mA h g^–1^. On the other hand, the development of SIBs has predominantly focused on overcoming the challenge of identifying suitable anode materials,^9^ as the commonly used graphite anode in Li-ion systems has a limited ability to intercalate Na-ion.^10^ Currently, one of the major challenges is finding viable materials that can effectively store both Li-ion and Na-ion to a significant extent in order to improve the electrochemical characteristics, such as specific capacity, cycling life and charge/discharge capability, which are crucial for promoting their practical use.

To date, numerous efforts have been made to improve the electrochemical efficiency of anode materials by nanostructuring the active materials with various shapes and sizes (for example, nanoparticles, nanowires, nanosheets, and nanocubes) to accommodate volume changes and shorten the ion diffusion paths.^11−13^ Over the past decade, two-dimensional (2D) layered materials have displayed potential and have garnered significant interest in both types of batteries. The unique structure of 2D materials enables faster ion diffusion compared to their bulk form, resulting in better rate performance and higher power density. Furthermore, progress has also been made in the exploration of nanocomposites consisting of quasi-2D platelets obtained from liquid phase exfoliation (LPE) of nonlayered materials and single-walled carbon nanotubes (SWCNTs) as potential anode materials for both Li-ion and Na-ion batteries, is due to their sturdy electrode structure, allowing them to achieve their theoretical capacity.^14,15^ The outstanding conductivity of the carbon nanotubes contributes to efficient charge distribution, thereby enhancing the specific capacity and rate performance.

Researchers have explored a wide array of electrode materials that can uptake both Li and Na-ions, including metal oxides (such as Co_3_O_4_ and NbO_2_),^16,17^ conversion reaction-based materials (sulfides and selenides),^18−20^ and alloying-based materials (Si, Ge and P).^21^ These electrodes have demonstrated good reversible capacity and cycling stability in both LIBs and SIBs. Although these materials still suffer from relatively low intrinsic conductivity, limiting their rate capability, combining them with conductive carbon materials (graphene, carbon nanotubes) can enhance electrical conductivity and buffer volume expansion.^11,22,23^ Despite significant advancements in anode materials for LIBs and SIBs, the search for new, high-performance anode materials remains a critical endeavour.

As a conversion-type electrode material, arsenic has a high theoretical capacity of 1073 mA h g^–1^ for both Li-ion and Na-ion storage, assuming the formation of Li_3_As^24^ and Na_3_As^25^ respectively, similar to the other pnictogen elements phosphorus, antimony, and bismuth.^26^ However, compared to these elements, arsenic has gained little attention in battery research due to valid concerns over its toxicity.^27−30^ Arsenic poisoning affects millions of people annually, primarily due to chronic exposure to naturally contaminated drinking water.^31^ Proper disposal of arsenic-containing batteries would be essential to prevent environmental contamination. However, arsenic compounds are already widely used as an additive in animal feed, in chalcogenide glasses for optical memory devices, in alloying electronic and photovoltaic substances, and in fabricating lead-acid batteries. Similarly, toxic elements such as lead, cadmium and cobalt are routinely used in lead-acid, nickel–cadmium and LIBs, respectively.^32^ Given the scarcity of literature on this topic, thoroughly investigating the potential of arsenic-containing materials as ion-storing electrode materials is important. Furthermore, in our recent report, we presented the application of As_2_S_3_/SWCNT composites as an anode for potassium ion batteries,^33^ demonstrating outstanding performance that competes with electrodes utilizing the As_2_S_3_ analogues Sb_2_S_3_ and Bi_2_S_3_ for KIB. Motivated by this, we investigated the utilization of As_4_S_4_ as an anode material for Li and Na-ion batteries.

Arsenic(II) sulfide (As_4_S_4_) is a nonlayered van der Waals material composed of discrete As_4_S_4_ molecules with one of two configurations, either realgar-type (R-type) or pararealgar (P-type). There are two realgar-type polymorphs (α- and β-As_4_S_4_), and two polymorphs composed of the pararealgar-type molecules, namely pararealgar and a synthetic phase known as As_4_S_4_(II).^34^ The R-type As_4_S_4_ molecule is sensitive to light with wavelength 500–670 nm, which causes a transformation to the P-type molecule via an As_4_S_5_ intermediate.^35^ As_4_S_4_, while still toxic, is considered relatively harmless compared to many other arsenic compounds, and it is commonly utilized in traditional Chinese medicine. Research conducted on mice has demonstrated that As_4_S_4_ is notably less toxic than As_2_O_3_, with a median lethal dose of 3.2 g kg^–1^ body weight, in contrast to 32–39 mg kg^–1^ for As_2_O_3_.^36^

As_4_S_4_ demonstrates a high theoretical capacity of 1253 mA h g^–1^ associated with the complete lithiation or sodiation of both arsenic and sulfur. However, to date, there has only been one study on its use as an electrode material, involving As_4_S_4_ (a mixture of α- and β-phases) and reduced graphene oxide composite electrodes as Li-ion storing anodes.^37^ The study predicted that molecular-cage-like clusters in the As_4_S_4_ represent the smallest anode units, offering independent intercalating routes and binding sites for accommodating Li-ion, similar to other metal chalcogenides. Hence, our objective is to expand on prior knowledge to maximize the capacity achieved by using electrodes composed of carbon nanotubes mixed with 2D platelets of As_4_S_4_.

While LPE is commonly employed in the production of 2D nanosheets from layered materials, its application to nonlayered materials is less common and more challenging.^38^ The layered structure facilitates easy conversion into nanosheets through liquid exfoliation, while nonlayered materials typically feature strong covalent or ionic bonds in three dimensions, posing a challenge for achieving anisotropic exfoliation. This often results in irregularly shaped quasi-2D platelets instead of 2D nanosheets.^38^ However, the quasi 2D-nanoplatelets produced from nonlayered materials have shown great potential as excellent electrode materials in Li/Na-ion batteries.^14,15,39^

In this paper, we explore the use of As_4_S_4_ quasi 2D-platelets as an anode material for both Li-ion and Na-ion battery anodes. We demonstrate that it is feasible to exfoliate As_4_S_4_in liquids to produce 2D platelets in substantial quantities, facilitated by the relatively weak van der Waals bonding between molecules. We thoroughly characterize the as-produced nanoplatelets using X-ray diffraction (XRD), X-ray photoelectron spectroscopy (XPS) and microscopic techniques (scanning electron microscopy (SEM), transmission electron microscopy (TEM) and atomic force microscopy (AFM)), reporting their morphology, structure, and stoichiometry. By employing single-walled carbon nanotubes as a binder, the 2D platelets of As_4_S_4_ can be readily solution-cast into films using filtration, making them suitable for use as anodes for both Li-ion and Na-ion batteries. Their electrochemical characteristics demonstrate a high capacity, which is close to theoretical values with commendable cycling stability.

## Results & Discussion

### LPE and Characterization of As_4_S_4_ Nanoparticles

**Figure 1:**
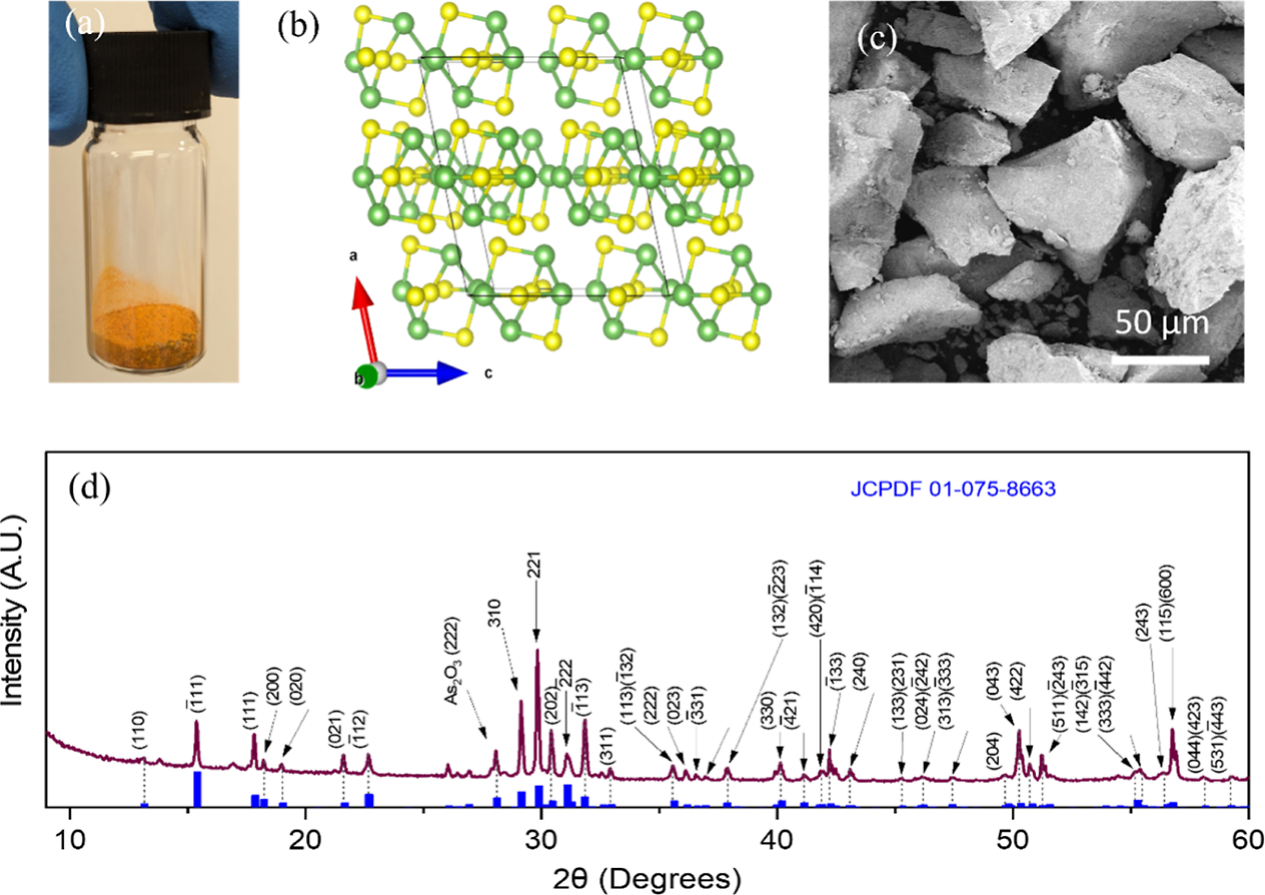
Structural characterization of bulk bonazziite (β-As_4_S_4_). (a) Photograph of bulk As_4_S_4_ powder. (b) Crystal structure of bulk As_4_S_4_. Arsenic atoms are depicted as green spheres, while sulfur atoms are indicated as yellow spheres. The black box in the structure represents the unit cell. (c) SEM image of the bulk As_4_S_4_ sample surface displaying nonlayered features. (d) XRD patterns of bulk As_4_S_4_ powder.

As_4_S_4_ (β-phase), a naturally occurring mineral known as bonazziite, belongs to the family of nonlayered materials.^40^ The As_4_S_4_ material was sourced commercially, and the received material was presented as a dark orange powder interspersed with brown chunks (Figure S1), which assumed a uniform dark orange hue upon grinding with a mortar and pestle (Figure 1a). The crystal structures of the α- and β-As_4_S_4_ possess the same molecular symmetry of *D*_2*d*_. However, they are arranged in different packings resulting in different space groups. The α-As_4_S_4_ phase possesses a monoclinic structure and crystallizes in space group *P*2_1_/*c*, containing four molecules located on-site C1 within the unit cell.^41^ The β-As_4_S_4_ consists of the same bonding molecules but with a more closely packed monoclinic structure in space group *C*_2_/*c*, and the unit cell contains two covalent As–S bonds and one metallic As–As bond per As atom in the primitive cell, C_2_ site symmetry (Figure 1b).^34^ As shown in Figure 1c, the SEM image of the bulk As_4_S_4_ powder surface revealed featureless particles with sizes ranging from 50 to 80 μm and confirmed the nonlayered morphology. XRD analysis was employed to identify the phase of the bulk material and to assess the crystallinity. As shown in Figure 1d, it confirmed that the commercial As_4_S_4_ powder was in the β-phase (PDF 01-075-8663).^42,43^ Small peaks corresponding to the (1 1 1) and (2 2 2) planes of As_2_O_3_ (PDF 01-071-2727) were detected in the bulk sample, suggesting some degree of photoinduced degradation had already taken place.^35^ Additionally, several peaks such as (1 1 1), (0 2 1), and (−1 1 2) were accompanied by smaller peaks at slightly lower 2θ angles. Lower angles correspond to larger planar spacings, so this is consistent with an expansion of the unit cell in parts of the sample. This was described by Bonazzi et al. as the initial stage in photodegradation where R–As_4_S_4_ molecules are randomly substituted by As_4_S_5_, leading to an anisotropic expansion of the unit cell parameters.^42−44^

#### Inert LPE

**Figure 2:**
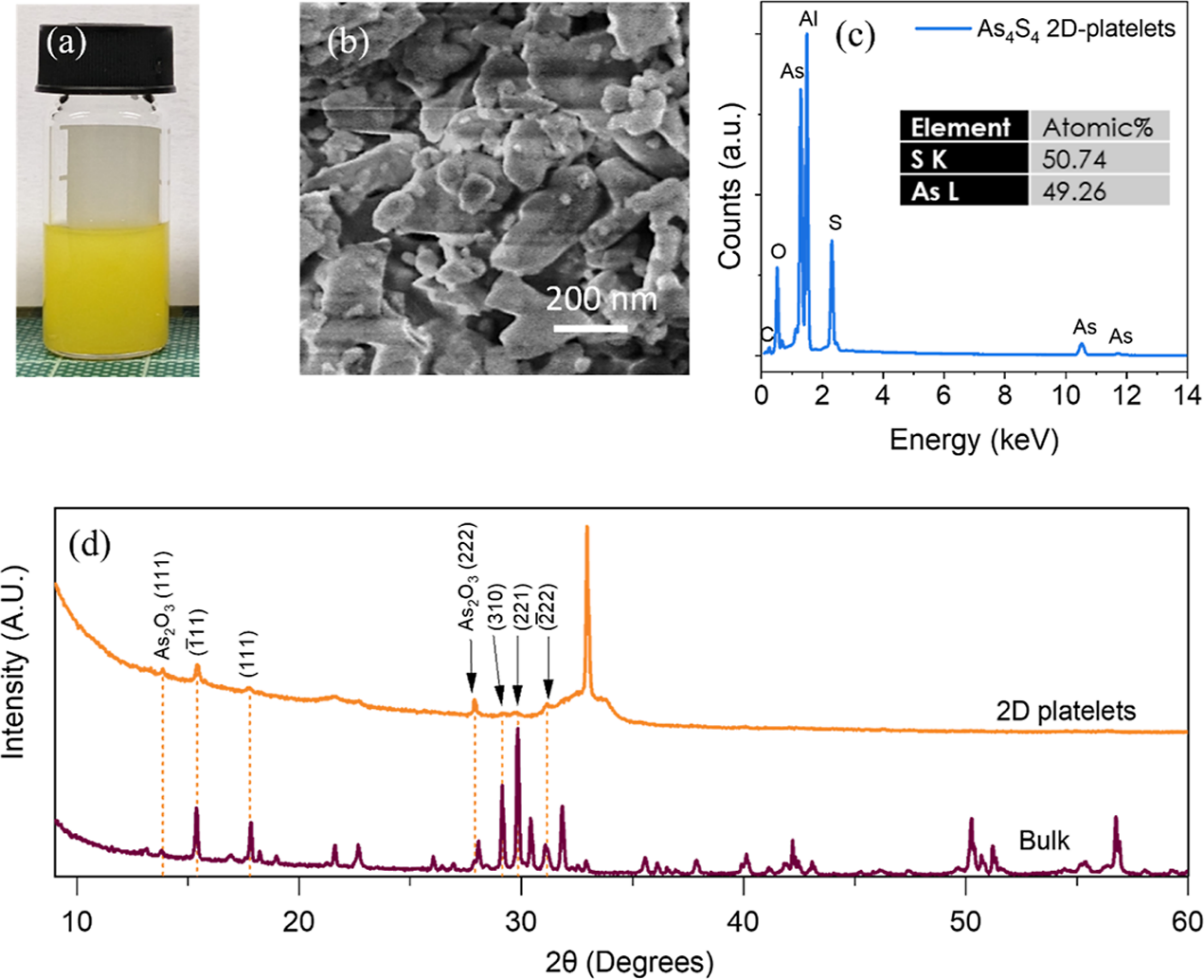
Characterization of LPE produced As_4_S_4_ material. (a) Photograph of the As_4_S_4_ 2D nanoplatelet dispersion after liquid exfoliation in an inert atmosphere. (b) SEM image of the 2D nanoplatelets. (c) SEM–EDX spectra of 2D nanoplatelets confirmed the presence of arsenic and sulfur elements. (d) XRD spectra of the bulk powder and the exfoliated 2D platelets.

As As_4_S_4_ does not have a layered crystal structure it is not expected that the application of liquid-phase exfoliation techniques will result in the formation of thin nanosheets. Instead, it is anticipated that a slight anisotropy in the van der Waals bonding strength between molecules could lead to the production of thick quasi-2D platelets with a low aspect ratio.^15,38^ In this study, the bulk β-As_4_S_4_ powder was converted into nanoplatelets by ultrasonication in isopropanol (IPA) for 12 h to produce stable nanoplatelet dispersions. Here, the benefits of using IPA as an appropriate sonication solvent are its nontoxicity, ability to create stable dispersions containing a significant amount of exfoliated material, and a relatively low boiling point in comparison to solvents like NMP or DMF.^45^ This makes it easier to remove for additional characterizations and processing of battery electrodes. The bulk β-As_4_S_4_ powder was bath-sonicated for 12 h in anhydrous (distilled and degassed) IPA under an N_2_ atmosphere to perform inert atmosphere LPE.^46^ The resultant dispersion was subjected to centrifugation at 400*g* in order to eliminate large unexfoliated material, and then centrifuged at 3800*g* to separate all 2D particles except the very smallest particles (see Experimental section). The sediment was subsequently dispersed in fresh IPA, resulting in a standard dispersion (Figure 2a) with a concentration of 1.3 mg mL^–1^, which was determined by vacuum filtration of the dispersion and subsequent mass weighing. This method of synthesis enables direct production of As_4_S_4_ nanoplatelets for the electrochemical storage of specific alkali ions, such as Li and Na.

#### Microscopic and Spectroscopic Characterization

In Figure 2b, the use of SEM showed that the material has a flat, 2D-platelet-like morphology, with lengths generally ranging between 100 and 500 nanometres. These results are consistent with 2D-flakes produced by LPE from other nonlayered materials.^14^ The sample chemical composition and stoichiometry were analyzed using energy dispersive X-ray spectroscopy (EDX) spectra. Figure 2c displays a sample spectrum indicating the presence of As, S, O, and Al. The surface oxygen contributes to the O signal, while the Al signal originates from the Al_2_O_3_-coated separator membrane. EDX-elemental maps display an even distribution of As and S atoms with an anticipated ratio of arsenic and sulfur stoichiometry of 1:1 (Figure S2). XRD was employed to identify the structural properties of the dispersed nanoparticles. In Figure 2d, the nanomaterial data only showed a small number of the strongest peaks of the β-phase, and specific highly intense reflections of bulk As_4_S_4_ such as (310) and (221) were notably absent in the exfoliated platelets. It is important to recognize that a few-layered exfoliated nanosheets are not expected to restack as perfectly as the bulk material.^47^ As a result, distinct XRD patterns are observed for 2D platelets compared to the bulk material. Additionally, exfoliated materials from nonlayered substances are predicted to generate amorphous nanoplatelets.^38^ Therefore, As_4_S_4_ is also expected to exhibit similar behavior. The noticeable decrease and disappearance of a few peaks indicate a significant reduction in crystallinity, suggesting that the material is predominantly amorphous following exfoliation. Additionally, a distinct peak at 33.02° in the nano As_4_S_4_ data was observed, which corresponds to the forbidden (2 0 0) reflection of the silicon substrate.^48^ The As_2_O_3_ peaks at (1 1 1) and (2 2 2) remained pronounced in the nanomaterial diffraction pattern. No peaks corresponding to the long-range crystal structure of pararealgar (PDF 01-083-1013) were detected in either the bulk or nanomaterial samples. This indicates that the bulk of the sample retained the original structure of the β-phase, possibly with a distribution of P-type As_4_S_4_ intermediate As_4_S_5_ molecules substituted for R-type As_4_S_4_ molecules.^44^

**Figure 3:**
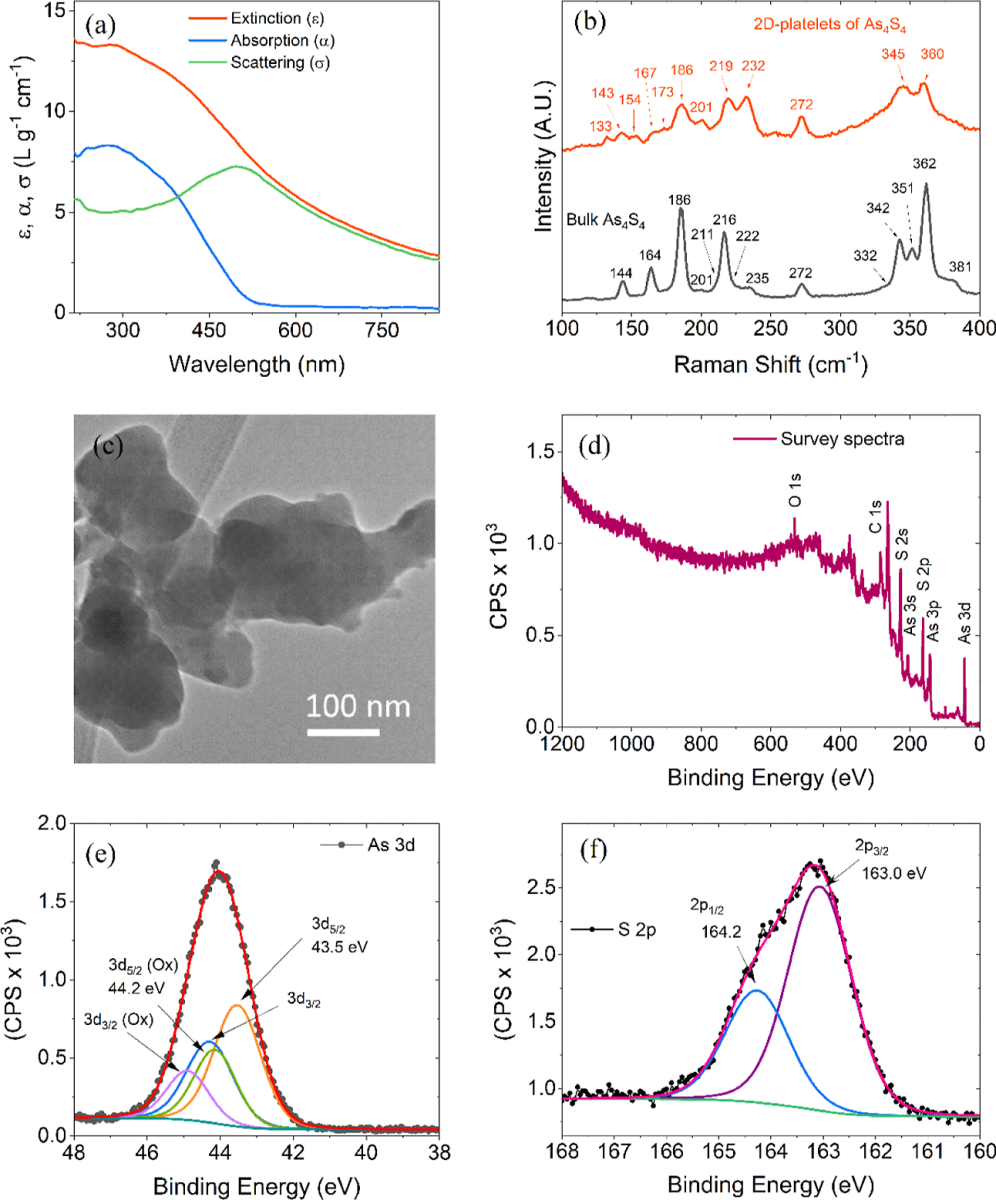
Structural characterization of As_4_S_4_ 2D nanoplatelets. (a) UV–visible extinction (ε), absorption (α), and scattering (σ) coefficient spectra of the 2D platelet dispersion. (b) Raman-scattering spectrum of bulk and 2D platelets. (c) Low magnification bright-field TEM image of As_4_S_4_ after exfoliation, confirming 2D platelet-like morphology. (d) XPS survey spectra of As_4_S_4_ 2D platelets. (e) High-resolution As 3d core level spectra. (f) Deconvoluted S 2p core level spectra of As_4_S_4_ 2D-nanoplatelets.

The investigation of the optical properties of the dispersed nanosheets utilized UV–visible spectroscopy, as depicted in Figure 3a. The extinction spectrum (Ext = −log *T*) was acquired and then transformed into an extinction coefficient spectrum (ε = Ext/*Cl*), where *l* represents the cuvette length. The scattering coefficients (σ) were measured using an integrating sphere by subtracting the absorption coefficient (α) from the extinction coefficient (ε) at each wavelength. In the α-absorption spectrum of the As_4_S_4,_ the band edge appears at a wavelength of ∼530 nm, corresponding to an optical gap of 2.34 eV. This is the first time the band gap of β-As_4_S_4_ nanostructures has been reported, and for comparison, no bandgap values have been reported for β-As_4_S_4_ in the literature.

The Raman spectra of bulk and exfoliated nanosheets of As_4_S_4_ are shown in Figure 3b. As previously mentioned, β-As_4_S_4_ can undergo a structural transformation when exposed to light within the 500–670 nm range, so to avoid this the spectra were acquired using a 785 nm wavelength laser. The spectrum obtained from the bulk sample closely matched the one reported for β-As_4_S_4_ by Muniz-Miranda et al., except for the shoulder at 236 cm^–1^ and peak at 272 cm^–1^.^49^ These are characteristic of P-type As_4_S_4_ molecules, indicating that some photodegradation had occurred on the sample surface. The spectrum from the nano-As_4_S_4_ sample exhibits pronounced features of both R-type and P-type molecules, indicating a greater degree of photodegradation had occurred compared to the bulk sample. P-type molecules have a shorter As–As bond length than the R-type molecule in β-As_4_S_4_ (2.51 Å vs 2.59 Å) resulting in an increased Raman shift. This explains the doubling of the peaks at 143, 167, and 186 cm^–1^ as both molecule types contribute to the spectrum.^49^ Similarly, the peak observed at 232 cm^–1^ is a distinctive feature of the bonding vibrations in As–As–As bonds, which exclusively occur in P-type molecules. The S–As–S bending mode, which occurs at approximately 219 cm^–1^ in the Raman spectrum of β-As_4_S_4_, indicates a cage structure and shifts to 272 cm^–1^ in the pararealgar phase. Meanwhile, the vibrational modes associated with the As–S bonds are nearly identical in both R-type and P-type As_4_S_4_ molecules, reflecting a consistent bond length of 2.24 Å in each molecule.^49^ Additionally, the structure of the exfoliated sheets was analyzed using TEM. In Figure 3c, the image shows the nearly 2D platelet-like structure of the exfoliated products, with typical lengths ranging in the hundreds of nanometers. AFM was employed to examine the length, thickness, and aspect ratio of the As_4_S_4_ platelets, and the findings are presented in Figure S3. The flakes have a thickness of ∼55 nm and lateral dimensions of ∼284 nm. The aspect ratio of the platelets was found to be ∼5.7, this small aspect ratio is attributed to the limited bonding anisotropy characteristic of nonlayered materials.

We performed XPS analysis on vacuum filtered films of 2D-nanoplatelets to gain a better insight into the chemical environment and electronic structure of the R-type As_4_S_4_. In Figure 3d, the XPS survey spectra indicate the existence of arsenic, sulfur, carbon, and oxygen. To correct the photoelectron line positions, a C 1s binding energy (BE) of 285.0 eV is used as a reference energy for charge correction. The spectrum in Figure 3e showed a single peak with a small shoulder in the As 3d spectrum. This resulted in a deconvoluted spectrum with two doublet components. The first doublet component displayed the main As 3d_5/2_ peak at 43.5 eV, representing 60% of the area, and 3d_3/2_ at 44.28 eV which is interpreted as coming from As atoms within the realgar (α or β-As_4_S_4_) matrix.^50−52^ The second doublet accounted for the remaining 40% of the area with the 3d_5/2_ at 44.14 eV and 3d_3/2_ at 44.9 eV. Previous research has indicated that As_2_O_3_ (arsenolite) and As_2_O_5_ a can be situated at about 46.1 and 44.4–45.1 eV BE, respectively.^53,54^ Consequently, the second doublet observed at high BE in the As 3d spectra was associated with small surface characteristics of arsenic oxide species, likely As_2_O_3_, present on the sample surface (see Figure S4 for O 1s). These results are consistent with the XRD and SEM observations for little surface oxidation of As_4_S_4_ after exfoliation. This apparent surface oxidation has commonly been reported for many arsenic-containing sulfides.^50,51^

Deconvolution of the S 2p spectrum was achieved using one pair of 2p spin–orbit peaks (Figure 3f), with fitted peaks positioned at 163 eV assigned to 2p_3/2_ and 164.2 eV assigned to 2p_1/2_. The observed S 2p peak positions are consistent with the literature values reported for the β-As_4_S_4_.^52^ The measured binding energies of As 3d for our 2D platelets are slightly different from the values reported in the literature for the realgar structure.^50−52^ This difference could be attributed to subtle changes in the chemical environment within the surface-bound As_4_S_4_ molecules after exfoliation. It seems that in these surface-bound molecules, the van der Waals forces are perturbed in comparison to those present in the bulk mineral matrix, and the perturbation appears to be significant enough to influence the As bonding chemistry in a realgar molecule. This apparent structural perturbation and the resultant features (As 3d_5/2_ at 43.8 eV) have also been reported by Pratt and Nesbitt for β-As_4_S_4_.^52^ Validation of these interpretations may necessitate additional research, potentially utilizing instrumentation based on synchrotron technology.

### Evaluation of Electrochemical Storage Properties of As_4_S_4_ Nanostructures as Anodes in Li-Ion & Na-Ion Batteries

In this study, we examine the performance of the liquid phase exfoliated As_4_S_4_ nanoplatelets as anode materials in both Li-ion and Na-ion battery systems. This allows us to demonstrate the practical applicability of these materials in the realm of energy storage. Additionally, we aim to compare their capabilities for storing lithium and sodium and hope to gain insights into the transport and storage mechanisms for these two types of ions. Furthermore, by examining the electrochemical behavior of the As_4_S_4_ nanoplatelets in battery electrodes, we can assess the quality and purity of the synthesized materials. Moreover, achieving near theoretical storage capacities with these materials for lithium and sodium ions would indicate the high purity of the material. This is because the theoretical capacity is determined by its elemental composition.

#### Fabrication of As_4_S_4_/SWCNT Nanocomposite Anodes

The battery electrode fabrication process typically involves applying the active material in nano form onto current collectors, accompanied by the addition of conductive additives and binders to form a composite electrode. However, this structure often leads to capacity fading over repeated cycles and exhibits capacities well below the theoretical value. This is typically attributed to factors such as the shape of the active particles, low electronic conductivity, significant volume expansion during the conversion process, and electrode damage during the charge–discharge process. In contrast, substituting traditional conductive additives and polymeric binders with SWCNTs has consistently demonstrated the ability to achieve high out-of-plane electrode conductivity, which helps in delivering charge effectively and maximizing capacity and rate capability. Additionally, the robust SWCNT network also allows the composite electrode to endure both morphological and volumetric changes.^22,55−58^ This successful electrode structure for storing lithium/sodium in the electrodes can also be applied to As_4_S_4_ active particles. However, to our knowledge, there is just one report of Li-ion electrodes fabricated from As_4_S_4_ nanostructures, and none of Na-ion electrodes. Here, we aim to assess the promise of nanocomposites consisting of As_4_S_4_ nanoplatelets produced via LPE and SWCNTs for producing high-performing lithium and sodium-ion anodes.

**Figure 4:**
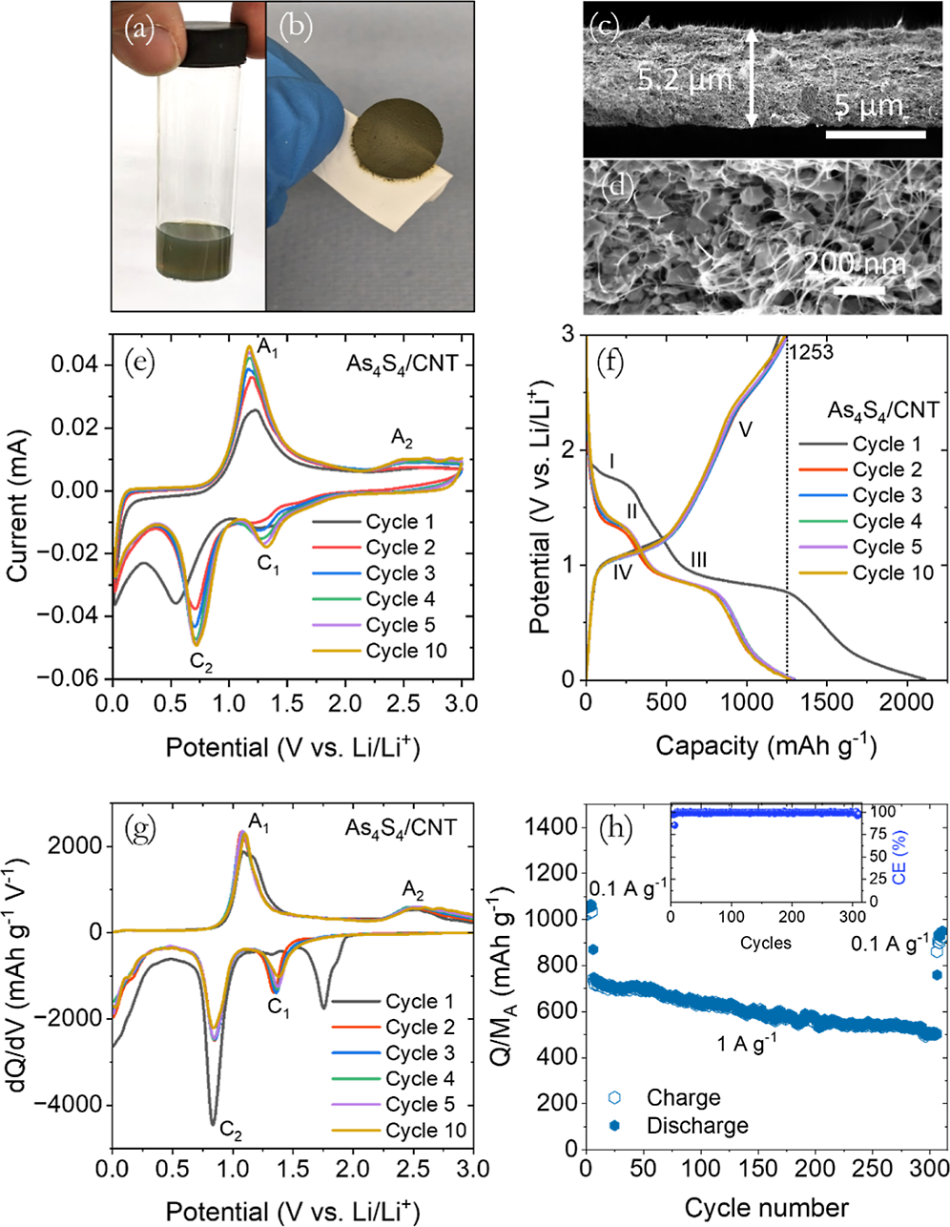
Li-ion storage characteristics of 2D-As_4_S_4_/SWCNT composite anodes (*M*_T_/*A* = 0.6 mg cm^–2^, *M*_f_^CNTs^ = 20%, *A* = 0.178 cm^2^). (a) Photograph of As_4_S_4_/SWCNT composite dispersion. (b) The photograph of a free-standing As_4_S_4_/SWCNT electrode. (c) SEM cross-sectional, and (d) top-view images of the composite electrode. (e) The first five consecutive cyclic voltammetry (CV) curves at a sweep rate of 0.1 mV s^–1^. (f) Galvanostatic charge–discharge voltage profiles for the first five and 10th cycles were collected at a current density of 0.1 A g^–1^, and (g) corresponding differential capacity plots. (h) Cycling performance of the composite electrode at a current density of 0.1 A g^–1^ for the first 6 cycles, followed by 300 cycles at a current density of 1 A g^–1^.

To prepare the nanocomposite electrodes, the dispersion of As_4_S_4_ nanoplatelets was mixed with a SWCNT dispersion in isopropanol (Figure 4a), and vacuum filtered to produce a film (please see the method section for detailed procedures). This process creates free-standing As_4_S_4_/SWCNT composite films (Figure 4b) containing 18–20 wt % of nanotubes without necessitating a polymeric binder. An areal mass loading of about 0.6 mg cm^–2^ was achieved. Subsequently, the films were cut into electrodes with an area of 0.178 cm^2^ for electrochemical testing. Figure 4c shows an SEM cross-sectional view of the As_4_S_4_/SWCNT electrode (total mass-loading 0.63 mg cm^–2^) revealing the electrode thickness of 5.2 μm. Figure 4d represents a closer view of the electrode surface with a uniform distribution of As_4_S_4_ nanoplatelets in a well-dispersed SWCNT network. Elemental composition maps of the electrode cross-section are displayed in Figure S5 of the Supporting Information, revealing the distribution of As and S atoms is evenly balanced, which aligns with the expected stoichiometry of As_4_S_4_. Here, the density of the electrode was calculated from mass loading and the thickness data, yielding a value of 1.22 g cm^–3^. Considering the densities of As_4_S_4_ (3.56 g cm^–3^) and SWCNT (1.8 g cm^–3^), the calculated porosity of the electrode is approximately 62%, falling within the reported range of values for 2D nanoplatelets.^22,33,58^

#### Evolution of LIB Performance of As_4_S_4_/SWCNT Nanocomposite Anodes

The electrochemical Li-storage properties of the As_4_S_4_/SWCNT nanocomposite electrodes were initially investigated using CV within the 0.01–3.00 V potential range. This experiment was carried out at a sweep rate of 0.1 mV s^–1^ in a half-cell configuration, using a fresh Li-disc as a reference. The As_4_S_4_/SWCNT cell had an open circuit voltage (OCV) of 2.69 V. In Figure 4e, the first discharge process (lithiation) showed a gradual increase in current starting at around 2 V, attributed to the lithiation of As_4_S_4_ to form Li_*x*_As_4_S_4_.^37^ At 1.34 V (C1), a noticeable broad cathodic peak is evident, primarily resulting from the conversion reaction that accompanies the formation of Li_2_S and amorphous As nanoparticles. Another peak was observed at 0.55 V (C_2_), probably due to the alloy reactions between Li^+^ and As that lead to the formation of Li_3_As, which is skewed by the formation of solid electrolyte interface (SEI).^30^ The overpotential associated with this reaction decreased in subsequent cycles and was later consistently observed at 0.72 V. During the charging process (delithiation), two anodic peaks were observed at 1.18 V (A_1_) and 2.4 V (A_2_) which were associated with dealloying and subsequent reversible conversion reactions, respectively. The CV curves in the subsequent scans displayed consistent shapes and completely overlapped, suggesting highly reversible electrochemical reactions.

Galvanostatic charge–discharge (GCD) experiments were performed on the As_4_S_4_/SWCNT electrodes in a half cell to validate the proposed mechanism. To start the initial activation processes, each electrode was subjected to 10 charge–discharge cycles at a current density of 0.1 A g^–1^. This standard procedure facilitates the development of a SEI layer on the surface of the electrode.^55^ The voltage profiles for the initial five and 10th cycles can be seen in Figure 4f, showing a voltage range of 0.01–3.0 V vs Li/Li^+^. In this study, the charge and discharge capacities, along with the current density, were determined using the mass of the active material (As_4_S_4_) and denoted as *Q*/*M*_A_. On the initial cycle, the electrode displayed a discharge capacity of 2110 mA h g^–1^, followed by a charge capacity of 1204 mA h g^–1^, resulting in a Coulombic efficiency (CE) of 57.1%. The charge capacity increased to 1255 mA h g^–1^ by the third cycle, effectively matching the theoretical capacity of 1253 mA h g^–1^, which is derived from the formation of Li_3.5_As and Li_2_S,^30^ which is significantly greater than the capacity associated with the formation of Li_3_As (1072 mA h g^–1^). Electrode CE was improved to 95.3% on the second cycle and gradually increased thereafter, reaching 97.7% by the 10th cycle. A charge capacity of 1240 mA h g^–1^ was obtained on the 10th cycle representing a modest decrease of 1.2% from that obtained on the third cycle. The charge–discharge curves maintained a stable shape in the following cycles, and the specific capacity remained almost the same, demonstrating the excellent stability of the nanostructures as anode materials. In Figure 4f, on the first discharge process, three plateau regions were observed in the voltage range of 2.0 to 1.7 V (marked as region I, denoting the intercalation process), 1.35 to 1.2 V (marked as region II, denoting the conversion process), and 0.9 to 0.75 V (marked as region III, denoting the alloying reaction). In the charging process, the plateau region between 1.0 to 1.2 V, is indicated as region IV represents reverse alloying, while the plateau region from 2.2 to 2.5 V (region V) indicates reverse conversion reactions. Except for the first discharge curve, the plateau regions of voltage profiles closely align with the peaks observed in the CV curves (Figure 4e).

To further study the Li-ion storage mechanism in the As_4_S_4_/SWCNT electrode, the differential capacity plots were examined by taking the derivative of the GCD curves (utilized to produce Figure 4f) and plotting dQ/dV against voltage. Figure 4g displays the differential capacity plots for the initial five and 10th cycles, which align with the following electrochemical reactions
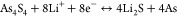
1a
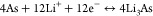
1b

Overall, this reaction is equivalent to
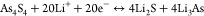
1c

The first discharge curve differs from the initial CV discharge curve. The processes involved in the first discharge cycle, including irreversible decomposition of the electrolyte, structural pulverization, and excessive lithium entrapment in the active material, may differ between the initial CV and GCD tests, because of the difference in the operational conditions. We have consistently observed this variation in lithium voltage during the first discharge cycle for various transition metal oxides and sulfide-based nanostructures used as anodes for Li-ion batteries.^16,22,55^ A distinct shoulder peak was seen at 1.86 V, followed by a larger peak at 1.76 V (Figure 4f). These peaks are believed to be associated with the adsorption of Li-ions onto the S atoms of the As_4_S_4_ molecule and the opening of the cage structure during the SEI formation on the surface of the As_4_S_4_/SWCNT electrode, as proposed by Kim et al.^37^ The position and absence of these peaks in subsequent cycles seem to be similar to the intercalation peaks observed in Sn_2_S_3_ anodes.^59^ The next noticeable peak at 1.46 V (C_1_) corresponds to the conversion reaction outlined in eq 1a. This reaction involves the production of amorphous arsenic nanoparticles through the reduction of As_4_S_4_ to As, and the formation of Li_2_S polysulfide. Another strong peak at 1.32 V (C_2_) corresponds to the formation of Li_3_As alloy outlined in eq 1b. In the anodic processes, the anodic peak at 1.0 V is attributed to the dealloy reaction, i.e., the electrochemical oxidation of Li_3_As to produce As. On the other hand, the oxidation peak observed at 2.4 V is attributed to a reverse conversion reaction, which involves the electrochemical oxidation of Li_2_S and arsenic to reform As_4_S_4_. This conversion process requires extensive structural reorganization due to the challenges of oxidizing S^2–^ in the highly stable Li_2_S material,^60^ making it difficult to reverse its chemical structure back to its original form, thereby impeding reversibility. As a result, the second discharge curve differs from the first, providing further confirmation that As_4_S_4_ is not fully recovered at the end of the charge (a comparable trend was noted in the CV curves depicted in Figure 4e). The remaining peaks are similar to those observed in the CV data, with slight shifts in position due to the different conditions imposed on the cell during GCD. However, their intensity has marginally decreased by the 10th cycle, reflecting the slight decrease in capacity. Notably, the conversion reduction peak has reduced in intensity while the corresponding oxidation peak appears to increase at the higher potential end. This additional capacity could be indicative of LiPS reduction at the anode, attributable to the undesired polysulfide shuttle effect.^60^

Following the initial activation cycles, we proceeded to cycle the electrode for 300 charge–discharge cycles at a higher current density of 1 A g^–1^. As shown in Figure 4h, throughout the cycling process, there was a slight decrease in charge–discharge capacities, reaching approximately 520 mA h g^–1^ with a CE of over 99% after the 300 cycles. At the 305th cycle, the current density was reduced to 0.1 A g^–1^. Subsequently, the As_4_S_4_/SWCNT electrode achieved a specific capacity of 950 mA h g^–1^ with a CE of over 98%. This demonstrates the electrochemical lithiation/delithiation reactions are reasonably reversible and exhibit satisfactory cycling performance. It is essential to evaluate the capacity contribution of the SWCNTs, using the experimental data demonstrated in Figure S6. The data reveals that the specific capacity of the SWCNT-only electrode in the Li-ion half-cell is approximately 300 mA h g^–1^ at a current density of 0.1 mA g^–1^, and for Na-ion is about 110 mA h g^–1^ at a current of 0.05 mA g^–1^. It is important to realize that the highest contribution of SWCNTs capacity in our As_4_S_4_/SWCNT electrodes is 60 mA h g^–1^ for Li-ion and 22 mA h g^–1^ for Na-ion. When compared to the overall capacity of the As_4_S_4_/SWCNT composite electrode, which is over 900 mA h g^–1^, the CNTs contributions are relatively small.

**Figure 5:**
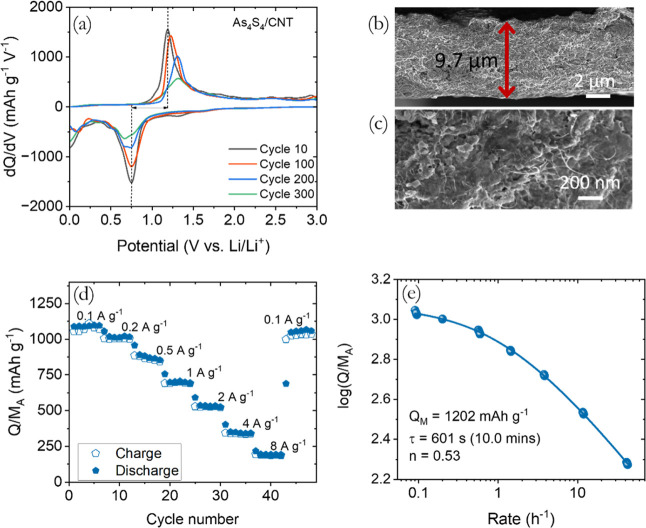
Postcycling and rate quantifying analysis for studying Li-ion storage properties. (a) Differential capacity plots were obtained for the 10th, 100th, 200th and 300th cycles while cycling at a current density of 1 A g^–1^. (b) SEM cross-section, and (c) close-view images of the As_4_S_4_/SWCNT composite electrode after 300 cycles. (d) The rate performance of the composite electrode at various current densities from 0.1 to 8 A g^–1^. (e) Specific capacity is graphed against the rate, *R* (*R* = (*I*/*M*_A_)/(*Q*/*M*_A_)) for the electrode. Equation 2 is used to fit the graph, and the figure displays the fitting parameters.

We further examined the differential capacity plots of As_4_S_4_/SWCNT electrodes for specific cycles (10th, 100th, 200th, and 300th cycle) at a current density of 1 A g^–1^ to investigate the redox reactions of As_4_S_4_ with Li-ions during cycling and detect any alterations in their electrochemical storage properties (see Figure 5a). The voltage hysteresis between alloy and dealloy reaction peaks gradually grew throughout the charge–discharge process, leading to significant irreversibility in the electrochemical charge storage and a progressive decline in Li-ion storage capacity, depicted in Figure 4h. Nevertheless, our composite electrodes displayed better cycling and rate performance compared to previous studies on As_4_S_4_/r-GO composites used as Li-ion anodes.

To examine the structure and morphology of the electrode after cycling, we used SEM cross-sectional imaging along with corresponding EDX elemental mapping, as shown in Figures 5b and S7. The SEM cross-sectional view of the As_4_S_4_/SWCNT electrode revealed an evident expansion from 5.2 μm for the uncycled electrode to 9.7 μm after 300 charge–discharge cycles. The electrode expansion after cycling suggests a decrease in density from 1.22 to 0.65 g cm^–3^, indicating an increase in porosity from 62% to 80% and internal surface area. Postcycled electrodes displayed a smoother surface with no visible 2D-platelets (Figure 5c), suggesting a consistent amorphous structure. The conversion-type materials generally experience a change in shape during cycling, making it highly improbable for them to maintain their original morphology after cycling. On postcycled electrodes, SEM–EDX elemental mappings reveal a uniform distribution of As and S atoms and arsenic and sulfur stoichiometry remain close to 1:1 (Figure S7). The rate capability was evaluated at various current densities ranging from 0.1 to 8 A g^–1^, as shown in Figure 5d. The data demonstrates fairly good stability and nearly complete restoration of the reversible capacity of 1060 mA h g^–1^ observed when the current density is switched from 8 to 0.1 A g^–1^ (cycles 43 to 48).

#### Understanding the Performance of Quantitative Rate Analysis

In previous studies,^61−63^ we have demonstrated that analyzing capacity versus rate data can provide valuable insights into the maximum achievable capacity of electrodes and the factors influencing their rate performance. We applied these methods to the data obtained from the As_4_S_4_/SWCNT nanocomposite electrodes to better understand the relationship between charge–discharge rate (*R*) and specific capacity, as shown in Figure 5e. The parameter *R*, representing charge–discharge rate, is defined as *R* = *I*/*Q*, where *I* is the specific current and *Q* is the specific capacity.^63,64^ This approach offers the advantage of using 1/*R*, we can measure the actual charging and discharging time of the electrode at a constant current. The information indicates that the measured capacity decreases as the rate (*R*) increases, consistent with general observations.^63,65^

For the analysis of this data, we employ a semiempirical fitting equation proposed recently by us^63^
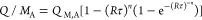
2

Here, fitting produces three fit parameters that describe the fit: *Q*_M,A_, representing the specific capacity (normalized to active mass) at an extremely low rate; τ, the distinctive charge/discharge time; and *n* is a parameter indicating whether diffusive (*n* = 0.5) or capacitive (electrical) (*n* = 1) limitations dominate the rate performance. As depicted in Figure 5e, eq 2 provides an excellent fit to the experimental data, yielding the following values for the fit: *Q*_M,A_ = 1202 mA h g^–1^, *n* = 0.53 and τ = 601 s. Each of these fit parameters will be discussed below.

The Q_M,A_ values indicate the maximum achievable capacity. Fitting of the As_4_S_4_/SWCNT electrode value data yielded of 1202 mA h g^–1^, which closely approximates the theoretical value of 1253 mA h g^–1^. This indicates that using 2D nanoplatelets of As_4_S_4_ with SWCNTs in place of binder and conductive additives enables almost complete utilization of the active material for Li-ion storage. This finding aligns with many prior studies on previous studies on electrodes based on different 2D materials.^22,55,56,58,66^ Here, the n value of 0.53 suggests that As_4_S_4_/SWCNT electrodes exhibit diffusion-limited behavior, especially for these thin electrodes (∼9.7 μm thick, after cycling). The time constant, τ, is an important parameter that indicates the minimum charge/discharge time required for the electrode to achieve 36.8% (1/*e*) of Q_M,Act_ and can be used to assess rate performance, where higher values of τ indicate poorer rate performance. Our measured τ-value of approximately 601 s implies moderate rate performance compared to previous battery materials based on various 2D platelets produced from nonlayered materials.^22,55^

To further analyze this, we note that, because of the dependence of τ on *L*_E_ (electrode thickness), a better parameter to consider is τ/*L*_E_^2^. Smaller values of τ/*L*_E_^2^ indicate better rate performance, with the very best performing electrodes displaying τ/*L*_E_^2^ ≈ 10^9^ s m^–2^.^63^ For our As_4_S_4_/SWCNT electrodes, τ/*L*_E_^2^ = 6.4 × 10^12^ s m^–2^ (where *L*_E_ ∼ 9.7 μm after cycling), falls within the range of values that have been recently reported for a range of other 2D Li or Na storing materials,^58,65^ indicating that the rate performance is consistent with the state-of-the-art for 2D materials.

#### Evolution of SIB Performance of As_4_S_4_/SWCNT Nanocomposite Anodes

**Figure 6:**
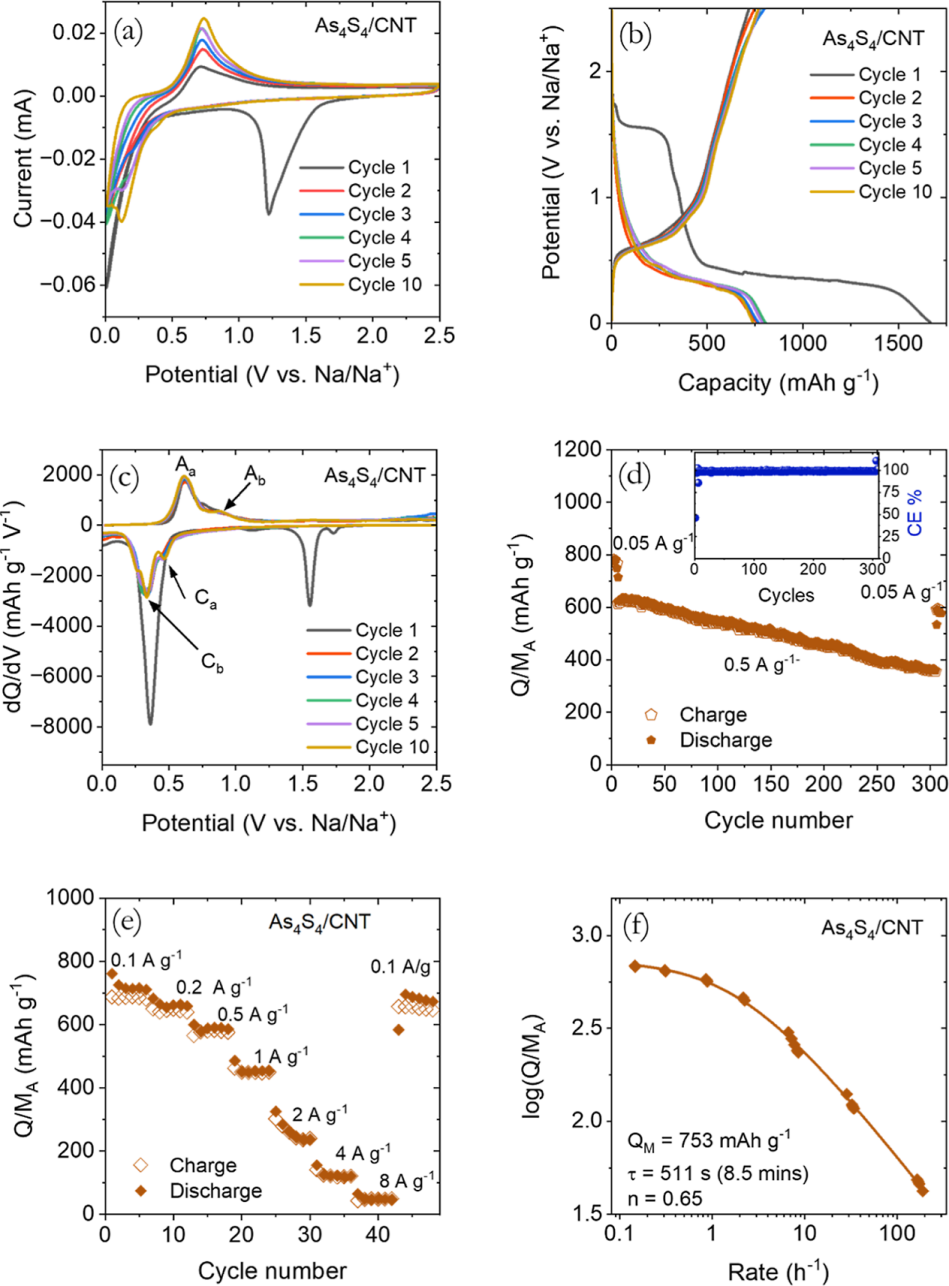
Na-ion storage characteristics of 2D-As_4_S_4_/SWCNT composite anodes. (*M*_T_/*A* = 0.6 mg cm^–2^, *M*_f_^CNTs^ = 20%, *A* = 0.178 cm^2^). (a) Cyclic voltammograms for the first 5 and 10th cycles at a sweep rate of 0.1 mV s^–1^. (b) GCD voltage profiles were collected for the first 5 and 10th cycles. (c) Corresponding differential capacity plots. (d) Charge–discharge cycling performance at 0.05 A g^–1^ for the initial 10 cycles and 0.5 A g^–1^ for the subsequent 300 cycles. (e) Rate performance of the electrode at various specific current densities from 0.1 to 8 A g^–1^. (f) The capacity versus rate curve was fitted to eq 2, and the figure displays the fitting parameters.

The As_4_S_4_ 2D-nanoplatelet/SWCNT composite electrodes were also examined as Na-ion battery anodes, replicating the previously conducted measurements for Li-ion batteries. The electrochemical Na-ion storage properties of these electrodes were first investigated using CV in a half-cell configuration with sodium foil as a reference. The CV curves were collected within a 0.01–2.5 V voltage range at a sweep rate of 0.1 mV s^–1^. Figure 6a presents the CV curves of the composite electrodes for the first 5 cycles. The As_4_S_4_/SWCNT cell displayed an OCV of 2.03 V. During the first cathodic sweep, a notable peak was divided into two separate peaks at 1.29 and 1.22 V, as a result of SEI layer formation, and the electrochemical generation of Na_2_S and the reduction of As_4_S_4_ to metallic arsenic. A gradual increase in current was observed from about 0.3 V before peaking sharply at 0.01 V, followed by a nearly identical pattern on the subsequent anodic sweep. The shape of this peak closely resembles those seen during sodium and lithium plating and stripping processes, possibly indicating Na^+^ deposition at the electrode surface. This could be due to significant overpotential resulting from high kinetic barriers to the alloying reaction when the electrodes were scanned at a sweep rate of 0.1 mV s^–1^. These characteristics are not evident when the electrodes are scanned at a very low current density of 0.05 A g^–1^ using GCD measurements (Figure 6b). It is important to realize that the dQ/dV plots collected from these voltage profiles (Figure 6c) show a noticeable difference from the CV curves, possibly due to variations in operational conditions, such as current density and batch-to-batch cell testing. Therefore, we assessed the redox chemistry of As_4_S_4_ with Na-ions by differential capacity plots analysis as shown in Figure 6c. For the first cycle, the discharge profile shows a shoulder peak at about 1.73 V attributed to the sodiation of As_4_S_4_ to form Na_*x*_As_4_S_4_. A prominent cathodic peak is visible at 1.56 V, mainly arising from an extra Na-ion adsorption/desorption during the generation of SEI on the As_4_S_4_/SWCNT electrode surface and some irreversible structural changes during the first discharge cycle. Therefore, the peak at 1.56 V is assigned to the generation of the SEI layer during the first discharge cycle and disappears in the subsequent scans. The subsequent noticeable cathodic peak at 0.46 V (*C*_a_) can be attributed to the conversion reaction, involving the electrochemical reduction of As_4_S_4_ to amorphous arsenic along with the formation of an amorphous Na_2_S matrix (eq 3a). Another strong peak at 0.33 V (*C*_b_) is probably attributed with the alloy reactions between Na^+^ and As, resulting in Na_3_As (eq 3b). The electrochemical redox reaction of As_4_S_4_ with Na can be described in the following two steps.
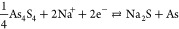
3a
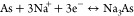
3b

The overall reaction can be represented as following
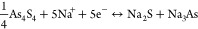
3c

The potential values observed for Na-ion anodes are not as positive as those seen in the CV plots for LIBs. As indicated in the literature, the usual charge–discharge potentials for common hosts are lower for sodium in comparison to lithium. This leads to slower reaction kinetics in SIBs because of the larger ionic radius of Na^+^, resulting in slower diffusion.^67,68^ In the anodic region, the peak observed at 0.62 V (*A*_a_) corresponds to the reverse alloying reaction while a small shoulder at 0.88 V (*A*_b_), is attributed to the reverse conversion reaction. It must be noted that the contribution of conversion reaction is minimal and is not fully reversible (eq 3a and that while a fully reversible reaction between Na and As_4_S_4_ predicts the theoretical capacity of 1253 mA h g^–1^ with the formation of Na_3_As,^30^ but which is higher than the experimental value the reversible reaction of only the arsenic component predict to yields a theoretical capacity of 752 mA h g^–1^. From the second cycle, the differential capacity plot exhibits consistent shapes and complete overlap, suggesting highly reversible electrochemical reactions.

The cycling performance of the As_4_S_4_/SWCNT nanocomposite electrodes was examined using a current density of 0.05 A g^–1^ for the first 10 cycles and 0.5 A g^–1^ for the following 300 cycles. The initial discharge and charge capacities in the first cycle are very high, approximately 1666 and 797 mA h g^–1^ (Figure 6b), respectively. This results in an initial CE of about 47.8%. As for the LIBs, the initial irreversible capacity loss of the nanocomposite electrode is attributed to the unavoidable decomposition of electrolytes and the creation of SEI.^22^ Furthermore, this low initial CE may be attributed to various factors such as the formation of irreversible phases like polysulfides, their shuttle effect, and the incomplete activation of the electrode material during its chemical or structural adjustments. Promisingly, ether-based electrolytes offer potential for achieving a high initial CE, and the utilization of specific molecular-designed phosphate-based electrolytes can greatly improve the reliability and stability of our electrodes.

After experiencing irreversible capacity losses in the initial cycles, As_4_S_4_/SWCNT composite electrodes achieved consistently high CE exceeding 98% as the cycling continued (inset of Figure 6d). During cycling at 0.5 A g^–1^, the charge capacities of As_4_S_4_/SWCNT linearly decreased from 626 mA h g^–1^ on the 10th cycle to 360 mA h g^–1^ on the 305th, it only shows 88% and 57% capacity retention after 100 and 300 cycles, respectively (compared to the first 0.5 A g^–1^ cycle). .

The rate performance was assessed at current densities ranging from 0.05 to 8 A g^–1^ as shown in Figure 6e. The composite electrodes exhibit the anticipated capacity decrease as the charge–discharge current increases due to both the capacity instability shown in Figure 6d and the increase in overpotential as the rate increases. For instance, when the current changed from 0.1 to 0.5 to 8 A g^–1^, the capacities observed were 715, 514, and 49 mA h g^–1^, respectively. Upon returning the current to 0.1 A g^–1^, the recovered capacity was 682 mA h g^–1^, indicating reasonable stability as the final capacity reached 95% of its previous value at 0.1 A g^–1^. The rate data was quantitatively analyzed in the same way as that obtained from the lithium half cells, yielding the following fitting parameters: *Q*_M,A_ = 753 mA h g^–1^, *n* = 0.65, τ = 511 s. Notably, the *Q*_M,A_ value almost exactly matches the theoretical capacity associated with the reversible sodiation of only the As component, suggesting that the conversion reaction was irreversible and did not contribute to the capacity. This also explains why the τ value was less than in the lithium half-cell implying better rate performance, as the conversion reaction has poorer kinetics than the arsenic alloying reaction.

To validate the difference in reversible capacity in SIB cells, we compared the redox behavior of As_4_S_4_ with Li-ion and Na-ion by analyzing the differential voltage plots during low-rate cycles before and after 300 cycles. In lithium-ion cells, the conversion reaction and subsequent alloying reaction were initially reversible, resulting in a stable and reversible capacity of 1202 mA h g^–1^, indicating the formation of Li_3.2_ As, which is larger than the generally accepted Li_3_As (1072 mA h g^–1^) alloy formation. However, the conversion reaction did not seem reversible in sodium-ion cells (as shown in Figures 6c and S8), with the measured capacities reflecting reversible charge storage only by the alloying reaction associated with only As, not S, resulting electrodes displaying reversible capacities of 753 mA h g^–1^, corresponding to the product of Na_2_As alloy, which is lower than the predicted capacity of 1253 mA h g^–1^ with the formation of Na_3_As similar to Li-cells. The reduced reversible capacity in sodium-ion cells might be attributed to mechanical pulverization of the electrode due to the larger ionic radius of Na^+^ compared to Li^+^, leading to large volume expansion (Figure S9).

## Conclusions

To summarize, we have demonstrated the successful LPE of nonlayered, bulk As_4_S_4_ material to produce quasi-2D platelets. The structure and stoichiometry of these platelets are confirmed by XRD, XPS, and SEM–EDX analysis, and align with that of bulk As_4_S_4_ structure. The combined AFM and TEM measurements indicated that the 2D platelets are thicker and possess an aspect ratio approaching ∼6. Finally, we characterized these As_4_S_4_ 2D-nanoplatelets as anodes for LIBs and SIBs by mixing them with carbon nanotubes. The As_4_S_4_/SWCNT anodes exhibited commendable performance for both Li-ion and Na-ion storage, with low-rate capacities of 1202 mA h g^–1^ at current density of 0.1 A g^–1^ for Li-ion batteries and 753 mA h g^–1^ at current density of 0.05 A g^–1^ for Na-ion batteries, and these experimental discharge capacities correspond to the products of Li_2.9_As & Li_2_S for Li-cells, and only Na_2_As alloy for Na-cells. A detailed quantitative rate analysis revealed a strong correlation between the highest achievable capacity (1202 mA h g^–1^) and the theoretical capacity (1253 mA h g^–1^), confirming the near full utilization of active material for Li-ion storage.

The As_4_S_4_ nanoplatelets are strong candidates for future use in LIBs and SIBs because of their impressive theoretical capacity, high efficiency, and significant capacity retention. In the future, research efforts can focus on addressing the challenges related to rate performance and striving to maximize the practical use of this material for energy storage purposes.

## Experimental Methods

### Materials & Exfoliation

Arsenic(II) sulfide powder (95%, 519111) and iso-propanol (HPLC with purity >99%) were purchased from Sigma-Aldrich. P3-SWNT were purchased from Carbon Solutions (carbonaceous >90%). The solvent was dried with a molecular sieve and distilled to remove impurities, then degassed by bubbling N_2_ overnight. All the electrolytes were purchased from Sigma-Aldrich.

The dispersion of As_4_S_4_ nanosheets was created using bath sonication (Branson CPX2800-E, 130 W) of the bulk As_4_S_4_ powder and the prepared solvent in a round-bottom flask under N_2_ atmosphere for 12 h. The sonic bath’s water was cooled by passing it through a heat exchanger that was placed in an ice bath. The dispersion was centrifuged at 400*g* for 2 h in a Hettich Mikro 220R centrifuge with a fixed-angle rotor with a radius of 100 mm. The resulting sediment, which contained large particles, was discarded, while the supernatant was put through a second round of centrifugation at 3800*g* for 2 h. After this, the supernatant containing very small particles was discarded, and the “size-selected” sediment was dispersed again in fresh iso-propanol, resulting in a standard dispersion of polydisperse nanoparticles.

### Nanomaterial & Composite Electrode Characterization

XRD analysis of the bulk and nanomaterial samples was conducted using a Panalytical X-ray diffractometer using a Cu K_α_ (λ = 1.5406 Å) radiation at 40 kV and 30 mA. The bulk sample was prepared by grinding the as-received material to a fine powder using a mortar and pestle and spreading it on a glass slide. The nanomaterial dispersion was drop-cast onto a 1 × 1 cm Si/SiO_2_ (100) wafer and heated to 60 °C to prepare the nanomaterial film.

Using a Zeiss Ultra Plus microscope SEM images were acquired at an accelerating voltage of 2 kV. The bulk sample was prepared by pressing an adhesive conductive carbon tab into a finely ground powder. Vacuum-filtered nanosheet film prepared on a Celgard membrane was used to acquire the SEM images. Samples for cross-sectional imaging of the composite electrodes were prepared by fracturing a fragment and mounting on a stub for imaging. TEM images were acquired using a JEOL 2100 microscope with an accelerating voltage of 200 kV. The standard dispersion was diluted to optical translucency, and then 10 μL were drop-cast onto C-coated Cu grids and let them dry overnight in a vacuum oven at 50 °C. The length of individual nanoparticles were measured one by one using ImageJ software and compiled to obtain representative statistical information. AFM measurements were performed with a Bruker multimode 8 microscope using an R3 cantilever (OLTESPA) operated in ScanAsyst mode. To prepare the sample, 10 μL of optically translucent dispersion was drop-cast onto Si/SiO_2_ substrates that were heated to 60 °C. The thickness of individual nanoparticles were measured using Gwyddion software and compiled to obtain representative statistical information.

A Renishaw inVia Qontor confocal Raman microscope with a 785 nm wavelength laser was used for Raman spectroscopy. The bulk measurements were performed on the as-received powder, and the nanomaterial sample was prepared by drop-casting 50 μL of the concentrated standard dispersion onto a Si/SiO_2_ substrate heated to 60 °C. Each spectrum was averaged over three accumulations with a 100× objective lens. An incident power of 1 mW was used to minimize the possibility of thermal damage to the samples. The UV–visible spectroscopic characterization was performed on a PerkinElmer Lambda 1050 spectrometer fitted with an integration sphere. The characterization sample was prepared by diluting the standard dispersion to optical translucency and placing 1 mL in a quartz cuvette with a path length of 4 mm. The extinction and absorption spectra were measured individually from 220 to 900 nm with an increment of 5 nm. The spectra of an IPA only sample were measured during the same session and subtracted from the dispersion spectra to isolate the nanomaterial contribution.

The XPS data were collected with an Omicron EA 125 Energy Analyzer using an Al K-alpha monochromated source at 1486.7 eV. High-resolution core-level components for C 1s, O 1s, S 3p, and As 3d were obtained using a pass energy of 20 eV. The spectra were obtained in high magnification mode with entrance and exit slits of 6 mm and 3 mm, respectively, resulting in an overall source and 0.6 eV instrument resolution. Survey spectra were collected using a 50 eV pass energy, high magnification mode, and entrance and exit slits of 6 mm each, resulting in an overall source and 1.5 eV instrument resolution. Each spectrum was subjected to a Shirley background deduction and was fitted using mixed Gaussian/Lorentzian peak shapes through an iterative least-squares method. Where multiple components were necessary to represent a single photoemission feature, the peak widths and shapes of the individual components were connected. However, the peak intensities and positions were allowed to vary independently. The analysis indicated that all C 1s features were found within 285.0 ± 0.5 eV with similar peak widths of 1.7–1.8 eV, suggesting consistent and minimal surface charging. Therefore, the adventitious C 1s feature was established at 285.0 eV as the reference for all reported energy data for charge correction of photoelectron line positions.

### Electrochemical Characterization

As_4_S_4_/SWCNT composite electrodes were prepared by vacuum filtering combined solutions of As_4_S_4_ 2D-nanoplatelets and SWCNTs in IPA. SWCNT dispersions were prepared by probe sonication of P3-SWNT (Vibracell CVX, 750 W, horn tip) and had a concentration of 0.1 mg mL^–1^. Ceramic coated separator (12 μm polypropylene coated with 2 μm AlO_*x*_ on each side, Cambridge Energy Solultions) was used as the filtration membrane. The electrodes have active mass loadings of As_4_S_4_ from 0.5 to 0.6 mg cm^–2^ with 18–20 wt % of SWCNTs. Half cells were set up in a 2032-type coin cell arrangement inside a glovebox that was filled with extremely pure Ar gas at O_2_ and H_2_O levels <0.1 ppm. In the lithium half cells, Li–metal discs (MTI Corp., 14 mm diameter) were used as the counter-electrode, and the separator was created from the same 12 μm PP ceramic-coated membrane that was used for filtration. The electrolyte was preprepared with 1 M lithium hexafluorophosphate (LiPF_6_) in ethylene carbonate/dimethyl carbonate (EC/DMC, 1:1, vol/vol) with added 10 wt % fluoroethylene carbonate (FEC) The sodium half cells were assembled with sodium metal counter-electrodes prepared from cubes stored under mineral oil (99.9% purity) and separators cut from 350 μm glass fiber membranes (Whatman GF 10). The electrolyte was 1 M sodium hexafluorophosphate (NaPF_6_, 98.0%, Fluorochem) in EC/DMC (1:1, vol/vol) with added 10 wt % FEC.

CV experiments were carried out with Biologic VMP-3 potentiostat-galvanostat at a sweep rate of 0.1 mV s^–1^. The lithium half cells were tested within a potential range of 0.01–3.00 V vs Li/Li^+^ while the sodium half cells were tested within a range of 0.01–2.50 V vs Na/Na^+^. Galvanostatic charge–discharge measurements were performed in the same potential ranges but using an Arbin galvanostat. The specific capacities and current densities were calculated using the active mass of As_4_S_4_ in the electrodes, and the CEs were derived from the ratio of the charge to discharge capacities.

## Supporting Information Available

The following file is available for free of charge. S1. The commercially received As_4_S_4_ powder. S2. SEM analysis of As_4_S_4_ 2D nanoplatelet filtered film. S3. Atomic force microscopy analysis of the exfoliated As_4_S_4_ 2D nanoplatelets. S4. XPS C 1s and O 1s core level spectra of As_4_S_4_ 2D nanoplatelet filtered film. S5. SEM analysis of the As_4_S_4_/SWCNT composite film. S6. The electrochemical performance of SWCNTs electrode for both Li-ion and Na-ion battery anodes. S7. Post-mortem SEM analysis of As_4_S_4_/SWCNT electrode after 300 GCD cycles for Li-ion storage. S8. Postcycling differential capacity plots for both Li and Na-ion storage anodes. S9. Post-mortem SEM analysis of As_4_S_4_/SWCNT electrode after 300 GCD cycles for Na-ion storage (PDF)
