# Supplementary material for: Liquid Processed Nano As4S4/SWCNTs Composite Electrodes for High-Performance Li-Ion and Na-Ion Battery Anodes
Source: Energy Fuels. 2024 Oct 15;38(21):21521–34. doi: 10.1021/acs.energyfuels.4c03525 (PMC11551956; doi:10.1021/acs.energyfuels.4c03525)
Supplement: Supplementary file 1 — ef4c03525_si_001.pdf [file ef4c03525_si_001.pdf]

Supporting Information

Liquid Processed Nano As_4S_4/SWCNTs Composite Electrodes for High-Performance Li-ion and Na-ion Battery Anodes

Mark McCrystall[1], Cian Gabbett[1], Harneet Kaur[1], Tian Carey[1], Jose Munera[1], Lee Gannon[1], Cormac Mc Guinness[1], Valeria Nicolosi[2], Jonathan N. Coleman[1]*, and Bharathi Konkena[1]*

[1]*School of Physics, CRANN & AMBER Research Centres, Trinity College Dublin, Dublin D2, D02 K8N4, Ireland*

[2]*School of Chemistry, CRANN & AMBER Research Centres, Trinity College Dublin, Dublin D2, D02 W9K7, Ireland*

Email: *Konkenab@tcd.ie (Bharathi Konkena); colemaj@tcd.ie (Jonathan N. Coleman)

CONTENTS:

S1. The commercially received As_4S_4 powder

S2. SEM analysis of As_4S_4 2D nanoplatelet filtered film

S3. Atomic force microscopy analysis of the exfoliated As_4S_4 2D nanoplatelets

S4. XPS C1s and O1s core level spectra of As_4S_4 2D nanoplatelet filtered film.

S5. SEM analysis of the As_4S_4/SWCNT composite film

S6. The electrochemical performance of SWCNTs electrode for both Li-ion and Na-ion battery anodes

S7. Post-mortem SEM analysis of As_4S_4/SWCNT electrode after 300 GCD cycles for Li-ion storage

S8. Post-cycling differential capacity plots for both Li and Na-ion storage anodes

S9. Post-mortem SEM analysis of As_4S_4/SWCNT electrode after 300 GCD cycles for Na-ion storage

S1. The commercially received As_4S_4 powder

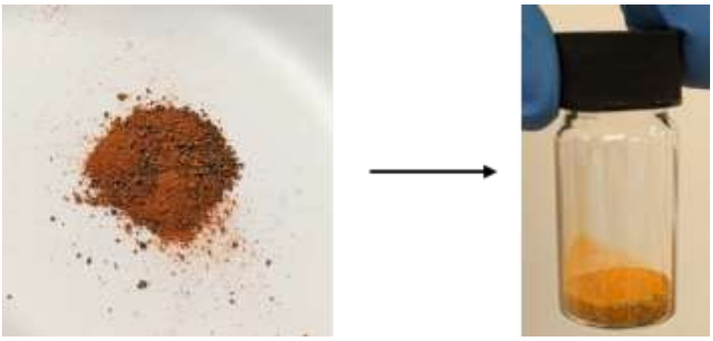

Figure S1. The commercially received As_4S_4 material and upon grinding.

S2. SEM analysis of As_4S_4 2D nanoplatelet filtered film

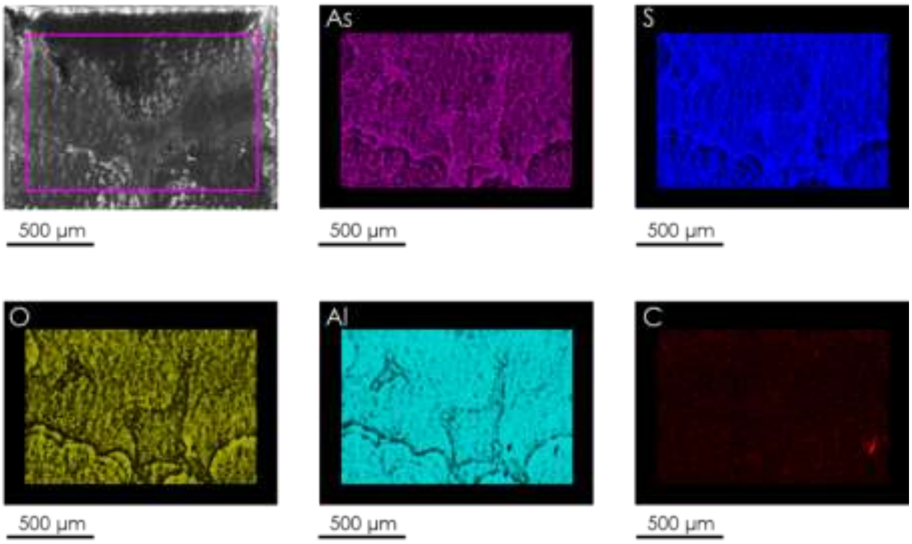

Figure S2. SEM cross-section image of the As_4S_4 2D nanoplatelet film with the corresponding elemental composition maps, for As, S, O, Al, and C. The surface oxygen contributes to the O signal, while the Al signal originates from the Al_2O_3-coated Celgard membrane. EDX spectra indicate the presence of As and S elements in the 2D platelet dispersion (Figure 2c). Elemental maps reveal a uniform distribution of As and S elements with an expected 1:1 arsenic and sulfur stoichiometry.

S3. Atomic force microscopy analysis of the exfoliated As_4S_4 2D nanoplatelets

Here, we employ atomic force microscopy (AFM) to analyze the thickness and aspect ratio (L/t) of the exfoliated nanoplatelets, and the findings are presented in Figure S3. Each nanoplatelet was measured for length and apparent thickness. The length and thickness distribution of approximately 12 nanoplatelets is illustrated in Figure S3, with average values indicated as <L> = 284±16 nm and <t> = 55±3 nm, along with standard errors. Furthermore, we determined the aspect ratio of each nanoplatelet by calculating the ratio of its length to thickness and displayed it as shown in Figure S3. The average aspect ratio is determined to be 5.7, which is considerably lower compared to the values ranging from 10 to 100 observed in layered materials.[1] However, this finding is consistent with measured aspect ratios for flakes produced by LPE from other non-layered materials.[2, 3] It has been suggested that low aspect ratios are anticipated in such materials due to low bonding anisotropy.[3, 4]

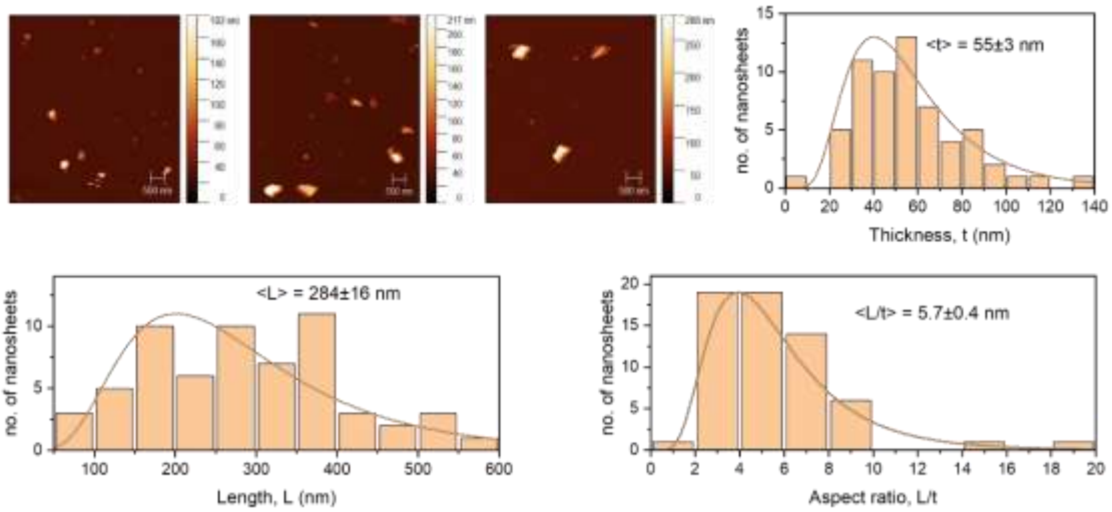

Figure S3. Atomic force microscopy image of As_4S_4 nanoplatelets deposited on a Si-substrate. Distribution plots illustrating the thickness, length and aspect ratio of nanoplatelets. Approximately twelve nanoplatelets were individually measured for length, width and height.

S4. XPS C1s and O1s core level spectra of As_4S_4 2D nanoplatelet filtered film

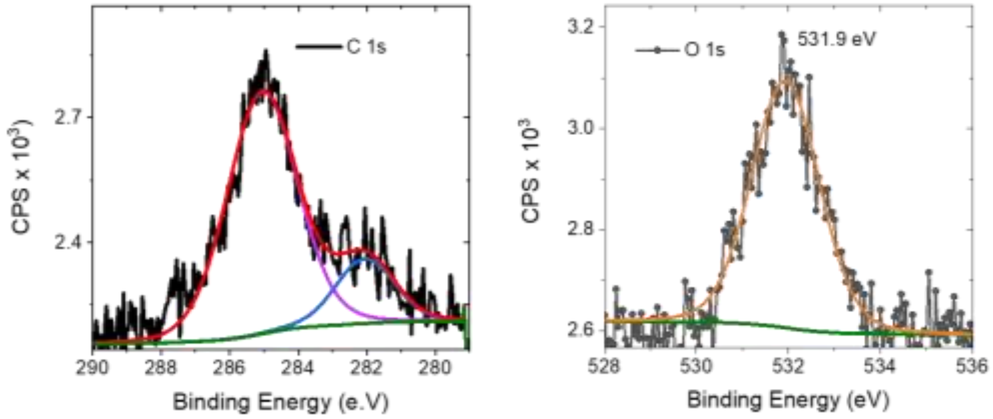

Figure S4. High-resolution C 1s and O 1s spectra of As_4S_4 2D nanoplatelets.

S5. SEM analysis of the As_4S_4/SWCNT composite film

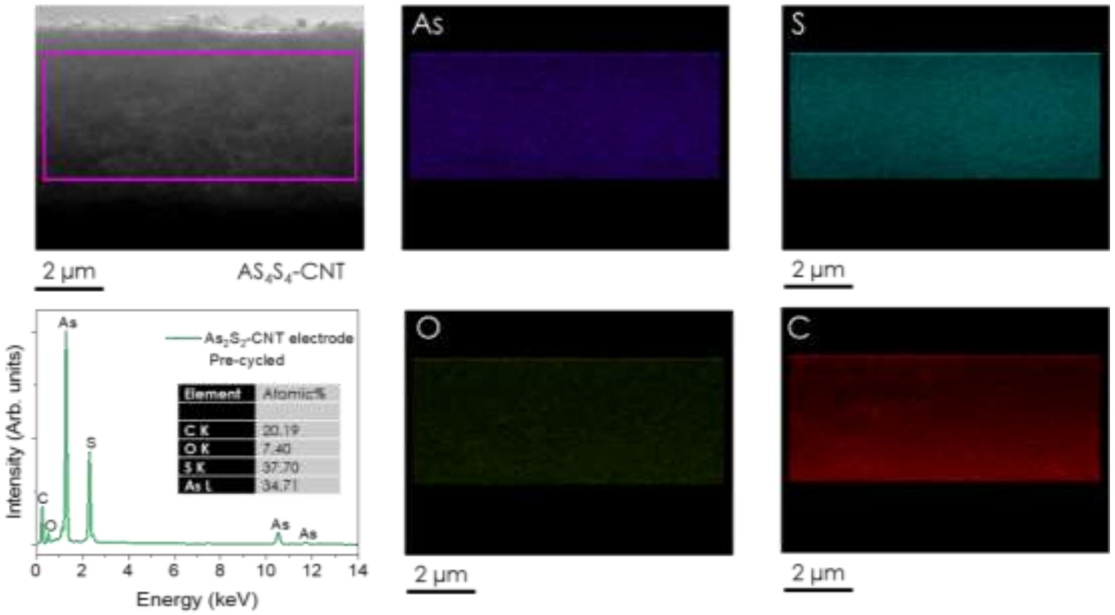

Figure S5. The SEM cross-section image of the As_4S_4/SWCNT composite film with the corresponding elemental composition maps, for As; S; O &C. The EDX spectra of the As_4S_4/SWCNT composite electrode, indicate the presence of As and S elements in the composite electrode. Elemental maps reveal a uniform distribution of As and S elements and remain close to 1:1 arsenic and sulfur stoichiometry.

S6. The electrochemical performance of SWCNT electrodes for both Li-ion and Na-ion battery anodes

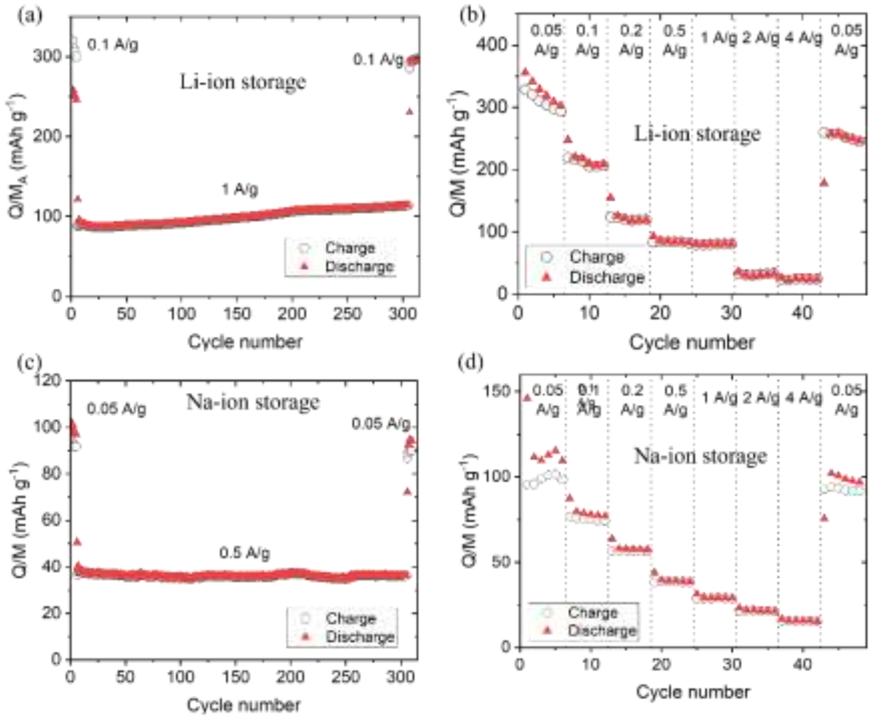

Figure S6. (a) Charge-discharge cycling at a current density of 0.1 and 1 A g^{-1}, and (b) Rate performance of SWCNTs film alone for Li-ion storage. (c) Charge-discharge cycling at a current density of 0.05 and 0.5 A g^{-1}, and (d) Rate performance of SWCNTs film alone for Na-ion storage.

S7. Post-mortem SEM analysis of As_4S_4@SWCNT electrode after 300 GCD cycles for use in Li ion-anode

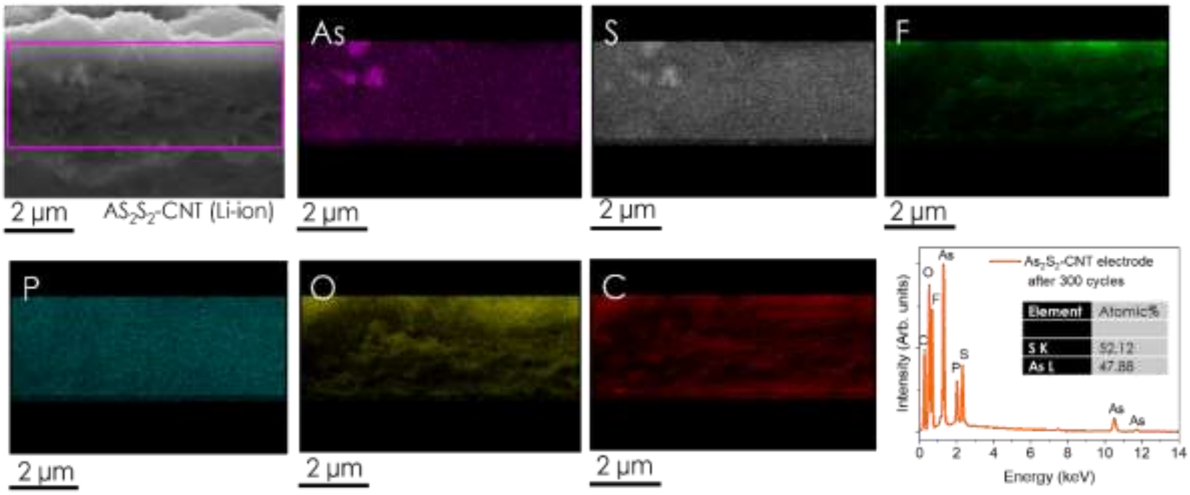

Figure S7. SEM cross-section image of the electrode after 300 charge-discharge cycles. The elemental mapping on the post-cycled electrode surface, for As, S, F, P, O and C. F and P originate from the electrolyte, while O is from electrode surface oxidation (from SEI and oxidation after washing the electrodes). EDX spectra on the post-cycled electrode represent the presence of uniformly distributed As and S elements. Elemental maps of the electrode revealed the stoichiometry of As and S remain close to 1:1.

S8. Post-cycling differential capacity plots analysis of As_4S_4/SWCNT electrode after 300 GCD cycles for use in both Li ion and Na-ion anode.

Here, we compared the redox behaviour of As_4S_4 with Li-ion and Na-ions by examining the differential capacity plots at low rate cycles before and after 300 cycles. Figure S8 illustrates the dQ/dV of low-rate cycles (at 0.1 A g^{-1} for Li-ion and 0.05 A g^{-1} for Na-ion) before and after 300 cycles of high-rate performance (1 A g^{-1} for Li-ion and 0.5 A g^{-1} for Na-ion storage). It is evident that the peak related to the conversion reaction disappeared in the lithium cell, while the sodium cell exhibited a similar pattern in pre and post cycling, due to the non-contributory nature of the conversion reaction to the capacity from the beginning. Due to this, only the alloy reaction related to the As nanoparticles contributes to the capacity (as shown in Eq. (3)) in the Na-ion storage process, resulting in a specific capacity close to the theoretical capacity of only As, which is 752 mAh g^{-1}, corresponding to the product of Na_2As alloy, which is lower than the predicted capacity of 1253 mAh g^{-1} with the formation of $Na_{3.5}As$.

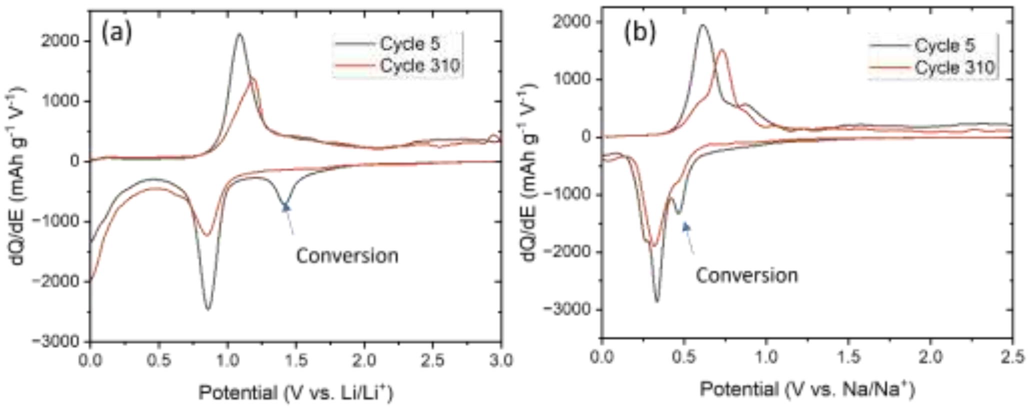

Figure S8. Post-mortem differential capacity (dQ/dV) plots of As_4S_4/SWCNT electrode low-rate cycles (at 0.1 A g^{-1} for Li-ion and 0.05 A g^{-1} for Na-ion) before and after 300 cycles of high-rate performance (1 A g^{-1} for Li-ion and 0.5 A g^{-1} for Na-ion storage).

S9. Post-mortem SEM analysis of As_4S_4/SWCNT electrode after 300 GCD cycles for Na-ion storage

SEM imaging was used to examine the morphological alterations in the post-cycled electrodes. After 300 cycles, it is evident that the electrode expanded from 5.2 µm for the uncycled electrode to 11.4 µm. This volume change is greater than that observed in a Li-ion storing anode due to larger size of Na-ion and the larger volume associated with the sodiated phases. EDX analysis of the electrode confirmed the presence of uniformly distributed As and S elements with the expected stoichiometry of As and S ~ 1:1. It was also observed that the As_4S_4/SWCNT composite electrode had a smooth surface with an amorphous texture.

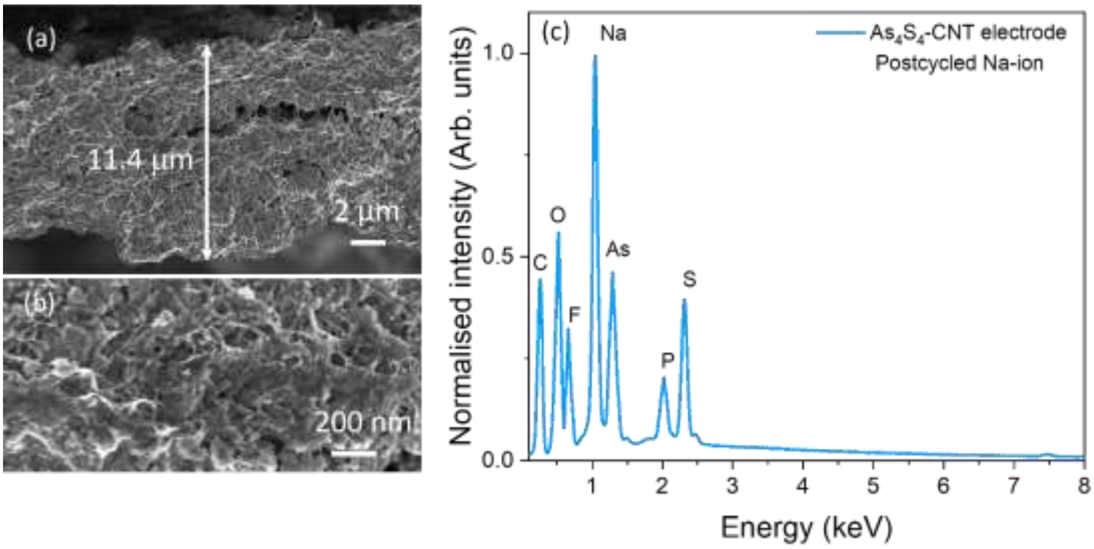

Figure S9. (a) Cross-section SEM image of the electrode, (b) a close-view image of the As_4S_4/SWCNT composite electrode after 300 charge-discharge cycles as a Na-ion storage anode, and (c) EDX spectrum of the post-cycled electrode.

References:

(1) Backes, C.; Campi, D.; Szydlowska, B. M.; Synnatschke, K.; Ojala, E.; Rashvand, F.; Harvey, A.; Griffin, A.; Sofer, Z.; Marzari, N.; et al. Equipartition of Energy Defines the Size–Thickness Relationship in Liquid-Exfoliated Nanosheets. *ACS Nano* **2019**, *13* (6), 7050-7061. DOI: 10.1021/acsnano.9b02234.
(2) Kaur, H.; Tian, R.; Roy, A.; McCrystall, M.; Smith, R.; Horvath, D. V.; Nicolosi, V.; Coleman, J. N. 2D nanosheets from fool's gold by LPE: High performance lithium-ion battery anodes made from stone. *FlatChem* **2021**, *30*, 100295. DOI: https://doi.org/10.1016/j.flatc.2021.100295.

(3) Kaur, H.; Tian, R.; Roy, A.; McCrystall, M.; Horvath, D. V.; Lozano Onrubia, G.; Smith, R.; Ruether, M.; Griffin, A.; Backes, C.; et al. Production of Quasi-2D Platelets of Nonlayered Iron Pyrite (FeS2) by Liquid-Phase Exfoliation for High Performance Battery Electrodes. *ACS Nano* **2020**, *14* (10), 13418-13432. DOI: 10.1021/acsnano.0c05292.
(4) Guo, Y.; Gupta, A.; Gilliam, M. S.; Debnath, A.; Yousaf, A.; Saha, S.; Levin, M. D.; Green, A. A.; Singh, A. K.; Wang, Q. H. Exfoliation of boron carbide into ultrathin nanosheets. *Nanoscale* **2021**, *13* (3), 1652-1662, 10.1039/D0NR07971E. DOI: 10.1039/D0NR07971E.
